# Quality Control Methods in Accelerometer Data Processing: Defining Minimum Wear Time

**DOI:** 10.1371/journal.pone.0067206

**Published:** 2013-06-24

**Authors:** Carly Rich, Marco Geraci, Lucy Griffiths, Francesco Sera, Carol Dezateux, Mario Cortina-Borja

**Affiliations:** Medical Research Centre of Epidemiology for Child Health, University College London, London, United Kingdom; McGill University, Canada

## Abstract

**Background:**

When using accelerometers to measure physical activity, researchers need to determine whether subjects have worn their device for a sufficient period to be included in analyses. We propose a minimum wear criterion using population-based accelerometer data, and explore the influence of gender and the purposeful inclusion of children with weekend data on reliability.

**Methods:**

Accelerometer data obtained during the age seven sweep of the UK Millennium Cohort Study were analysed. Children were asked to wear an ActiGraph GT1M accelerometer for seven days. Reliability coefficients(*r*) of mean daily counts/minute were calculated using the Spearman-Brown formula based on the intraclass correlation coefficient. An *r* of 1.0 indicates that all the variation is between- rather than within-children and that measurement is 100% reliable. An *r* of 0.8 is often regarded as acceptable reliability. Analyses were repeated on data from children who met different minimum daily wear times (one to 10 hours) and wear days (one to seven days). Analyses were conducted for all children, separately for boys and girls, and separately for children with and without weekend data.

**Results:**

At least one hour of wear time data was obtained from 7,704 singletons. Reliability increased as the minimum number of days and the daily wear time increased. A high reliability (*r* = 0.86) and sample size (*n* = 6,528) was achieved when children with ≥ two days lasting ≥10 hours/day were included in analyses. Reliability coefficients were similar for both genders. Purposeful sampling of children with weekend data resulted in comparable reliabilities to those calculated independent of weekend wear.

**Conclusion:**

Quality control procedures should be undertaken before analysing accelerometer data in large-scale studies. Using data from children with ≥ two days lasting ≥10 hours/day should provide reliable estimates of physical activity. It’s unnecessary to include only children with accelerometer data collected during weekends in analyses.

## Introduction

Children’s physical activity (PA) is a difficult behaviour to measure as it is sporadic, intermittent and characterised by substantial inter- and intra-individual variation [1]. In recent years accelerometers have been regarded as the ‘gold standard’ method to examine PA in childhood populations [2]. Children are asked to wear their accelerometer for a fixed period of time, typically during all waking hours for seven consecutive days [3], [4], [5]. Despite various incentives and reminders, children rarely wear their accelerometer for this entire period. As a result, researchers need to determine whether each child wore their accelerometer for long enough to provide a reliable estimate of PA and be included in analyses. This can be achieved by defining the minimum number of minutes per day and the minimum number of days that the accelerometer needs to be worn by each child.

Reliability determines the consistency of a set of measurements or of a measuring instrument [6]. The duration of daily wear time must be long enough to remove days when the accelerometer was not worn but short enough to prevent unnecessary days being removed from analyses, and the number of days the accelerometer needs to worn by each child must provide a reliable estimate of children’s habitual PA. No single value has been used by large-scale studies in children to define the minimum daily wear time: thresholds have ranged from at least four [7] to at least 10 [3], [4], [5], [8], [9] hours per day. Large-scale accelerometer studies in children have also used a range of thresholds to define the minimum number of wear days required by each child to be included in analyses, although at least three days per child has been most commonly used [4], [5], [10], [11].

Given recent evidence that children’s PA varies according to the time of day and day of the week [12], it is necessary to determine the minimum daily wear time and the number of wear days required to reliably estimate children’s habitual activity. No studies have explored the influence of varying the minimum daily wear time threshold on the reliability of accelerometer-determined PA measurement in pre-pubertal primary school-aged children. In those available, reliability estimates in preschool [13], [14] and older children [15] have been investigated. Previous research has shown that the child’s age influences the minimum number of accelerometer wear days required to reliably estimate PA; it is therefore likely that the minimum daily wear time is dependent on age. Researchers have looked at the influence of varying the minimum number of days required by each child to be included in analyses on the reliability of PA measurement but their findings are inconsistent, and study populations tend to be geographically clustered [16], [17], [18].

There are substantial gender differences in children’s PA [5], [8], [11]; however, previous research on the influence of varying the thresholds used to define minimum wear time have combined data from boys and girls. Although findings are inconsistent, previous research also found gender differences between children who did and did not provide reliable data in children’s accelerometer studies [3], [11], [15], [19]. Furthermore, studies have found that children’s PA varies between weekdays and weekend days [12]. Despite this, few studies [8], [12], [20] have considered whether or not children with week and weekend wear days are required to reliably estimate habitual PA.

Given the impact that these data processing procedures can have on derived activity variables and lack of previous research in pre-pubertal primary school aged children, further clarification on data cleaning methods is needed for researchers using these devices [21], [22], [23]. Esliger *et al* [24] emphasised the need for studies to evaluate within- and between-day variations in PA and in particular how these vary by gender. The aim of this study was to propose a threshold for the minimum number of hours per day and the minimum number of days of data required from each child to achieve reliable estimates of PA in population-based accelerometer studies. The influence of gender and the purposeful inclusion of children with and without weekend day data was also explored.

## Methods

### Study Population

We analyse population-based accelerometer data obtained as part of the Millennium Cohort Study (MCS). The MCS is a longitudinal UK-wide prospective study of children born in the new century sampled to ensure an adequate representation of all four UK countries, disadvantaged areas, and ethnic minority groups [25]. At age seven years, accelerometers were used to measure children’s PA levels. All children were invited to wear an accelerometer and written consent was obtained from parents/guardians of those agreeing.

### Accelerometer Protocol

Activity was measured using the ActiGraph GT1M (ActiGraph, Florida, USA), a small (3.8×3.7×1.8 cm), lightweight (27 g) uni-axial accelerometer that measures volumes and patterns of activity. The ActiGraph has been extensively validated in children [26], [27], [28], and is robust when used in large-scale studies in children [3], [4], [5], [8]. A 15-second sampling epoch was selected in order to optimize the ability to capture the sporadic nature of children’s activity [1]. Children were asked to wear the accelerometer on an elasticated belt on the right hip for seven consecutive days during all waking hours, except during bathing or swimming. Accelerometers were posted to families who were asked to return it as soon as possible after the monitoring period using a supplied pre-paid envelope. Accelerometers were distributed between May 2008 and August 2009.

Ethical approval for the MCS accelerometer study was granted by the Northern and Yorkshire Research Ethics Committee (REC number: 07/MRE03/32). The MCS data for surveys 1 to 4 are currently available via the Economic and Social Data Service; the MCS accelerometer data will be also be available shortly at the beginning of 2013.

### Statistical Analyses

Accelerometer data were downloaded using ActiLife Lifestyle Monitoring software (version 3.2.11) and processed using algorithms developed in R (version 2.14.1) [29]. Accelerometer non-wear was defined as any time period of consecutive zero-counts for a minimum of 20 minutes [8]. Data from all singleton children who returned an accelerometer with at least one hour of wear time data (periods in which non-wear was not identified) were eligible for inclusion in our analyses (*n* = 7,704). Twins and triplets were not included in the analyses because data were unintentionally not coded to allow the interview and accelerometer data for twins and triplets to be accurately linked.

All analyses were repeated using different samples depending on whether children met the varying threshold used to define wear time based on the minimum daily wear time (one to 10 hours) and the minimum number of wear days (one to seven days). Analyses were conducted for boys and girls combined, and separately. Analyses were also repeated separately for children that did, and did not, have at least one weekend wear day (of at least 10 hours wear). This wear time period was chosen because it is most often used by large-scale studies in children to define the minimum daily wear period [3], [4], [5], [8], [9].

The reliability of accelerometer-determined mean daily counts per minute (cpm) was calculated using the Spearman-Brown prophecy formula [30], [31] based on the intraclass correlation coefficient (ICC) as a measure of reliability. The distribution of mean daily cpm was skewed so the Box-Cox family of transformations were used to account for non-normality [32]. The asymmetry parameter in this family was chosen by maximising the profile log-likelihood using the R function boxcox [33]. A linear mixed-effects (LME) model was fitted to the transformed cpm using the MCS survey and non-response weights to account for the clustered sampling and attrition between contacts [34]. Single day ICC were calculated from the fitted LME models with the R function ICC1.lme [35]. The ICC describes how strongly units in the same group resemble each other, and is defined as the ratio of between-individual variance to the sum of the between- and within- individual variance [15]. The ICC is the most common way of summarizing the consistency of measurement across days [36]. An ICC value of 1.0 indicates that all the variation is between- rather than within-children, corresponding to perfect reliability or repeatability. An ICC value of 0.8 is commonly regarded as a marker of acceptable reliability.

Single-day ICC values were then used to calculate the influence of shortening or lengthening the monitoring period on the reliability of PA-measurement using the Spearman-Brown prophecy defined in the following equation:
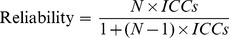
where: *N* = the number of days required, *ICCs* = single-day reliability [37]. We used heatmaps developed in R to produce graphical representations of reliability trends by minimum daily wear time and minimum days of wear day time.

## Results

### Total Sample

A total of 13,681 singleton children were interviewed at age seven years in the MCS: 12,872 (94.1%) of these parents/guardians gave consent for their child to wear an accelerometer. Accelerometers were sent to 12,303 (89.9%) consenting singletons; 27 (0.2%) singletons were not sent an accelerometer because the fieldwork team were unable to send it during the requested time period, and full contact details of the remaining 542 (4.2%) singletons were unavailable. A total of 9,772 singletons returned an accelerometer, of which 1,106 parents/guardians explicitly stated that the accelerometer had not been worn. A total of 7,704 (59.9% of consenting singletons) singleton children returned an accelerometer with at least one hour of wear time data (Table 1). There were 5,878 children with files that contained at least one hour of wear time data for greater than seven days and who had presumably worn the accelerometer for longer than the wear time period requested.

**Table 1:** Number of children included in analyses according to minimum daily wear time and minimum number of wear days for the total sample and by gender (boys, girls).

	≥1 hour		≥2 hours		≥3 hours		≥4 hours		≥5 hours		≥6 hours		≥7 hours	
≥1 day	7704		7579		7499		7431		7323		7184		7110	
	3815,	3889	3738,	3841	3694,	3805	3667,	3764	3601,	3722	3520,	3664	3472,	3638
≥2 days	7454		7370		7258		7120		6985		6916		6865	
	3675,	3779	3631,	3739	3583,	3675	3503,	3617	3417,	3568	3376,	3540	3356,	3509
≥3 days	7153		7000		6894		6820		6755		6708		6657	
	3493,	3660	3419,	3581	3364,	3530	3321,	3499	3292,	3463	3269,	3439	3237,	3420
≥4 days	6898		6775		6703		6635		6569		6504		6442	
	3364,	3534	3309,	3466	3270,	3433	3240,	3395	3208,	3361	3171,	3333	3141,	3301
≥5 days	6704		6590		6527		6452		6358		6269		6171	
	3268,	3436	3210,	3380	3181,	3346	3148,	3304	3104,	3254	3058,	3211	3016,	3155
≥6 days	6510		6399		6316		6205		6080		5898		5677	
	3172,	3338	3125,	3274	3088,	3228	3040,	3165	2984,	3096	2899,	2999	2794,	2883
≥7 days	6282		6146		6020		5781		5411		4951		4504	
	3061,	3221	2996,	3150	2953,	3067	2841,	2940	2667,	2744	2453,	2498	2246,	2258
≥8 days	5878		5650		5323		4596		3252		1902		1062	
	2878,	3000	2781,	2869	2633,	2690	2278,	2318	1632,	1620	958,	944	523,	539
≥9 days	5309		4728		3620		2090		857		335		177	
	2612,	2697	2327,	2401	1789,	1831	1048,	1042	427,	430	178,	157	95,	82
≥10 days	2891		1706		868		412		176		91		67	
	1422,	1469	845,	861	442,	426	208,	204	97,	79	53,	38	40,	27
	**≥8 hours**		**≥9 hours**		**≥10 hours**		**≥11 hours**		**≥12 hours**		**≥13 hours**		**≥14 hours**	
≥1 day	7031		6964		6853		6647		5955		4136		1003	
	3442,	3589	3409,	3555	3351,	3502	3250,	3397	2942,	3013	2087,	2049	1140,	1003
≥2 days	6784		6677		6528		6142		4841		2449		872	
	3315,	3469	3262,	3415	3188,	3340	3023,	3119	2443,	2398	1294,	1155	493,	379
≥3 days	6567		6408		6181		5513		3716		1421		392	
	3203,	3364	3132,	3276	3021,	3160	2749,	2764	1919,	1797	794,	627	218,	174
≥4 days	6327		6120		5776		4768		2722		802		216	
	3083,	3244	2989,	3131	2845,	2931	2428,	2340	1451,	1271	437,	365	122,	94
≥5 days	5993		5689		5033		3738		1750		438		129	
	2932,	3061	2801,	2888	2528,	2505	1932,	1806	940,	810	240,	198	67,	62
≥6 days	5337		4807		3903		2456		883		232		86	
	2634,	2703	2402,	2405	1993,	1910	1283,	1173	473,	410	134,	98	44,	42
≥7 days	3972		3202		2177		1077		327		100		37	
	2000,	1972	1657,	1545	1156,	1021	594,	483	187,	140	56,	44	19,	18
≥8 days	625		381		214		111		49		27		13	
	319,	306	205,	176	119,	95	64,	47	28,	21	15,	12	6,	7
≥9 days	111		76		45		32		20		13		8	
	61,	50	44,	32	27,	18	19,	13	11,	9	7,	6	3,	5
≥10 days	46		33		28		19		15		8		6	
	26,	20	19,	14	16,	12	11,	8	8,	7	4,	4	2,	4

The reliability of PA measurement was influenced by the minimum daily wear time and the minimum number of days of data required by each child for inclusion in analyses (Table 2). Reliability coefficients increased as the minimum number of days required by each child for inclusion in analyses increased (between one to ten days) and also increased as the minimum daily wear time increased from at least one hour per day up to, but no more than, at least eight hours per day.

**Table 2:** Reliability coefficients derived according to minimum daily wear time and minimum number of wear days (total sample).

	≥1 hour	≥2 hours	≥3 hours	≥4 hours	≥5 hours	≥6 hours	≥7 hours	≥8 hours	≥9 hours	≥10 hours	≥11 hours	≥12 hours	≥13 hours	≥14 hours
≥1 day	0.36	0.36	0.40	0.50	0.61	0.69	0.74	0.76	0.76	0.76	0.73	0.66	0.57	0.51
≥2 days	0.53	0.53	0.57	0.67	0.76	0.82	0.85	0.86	0.86	0.86	0.84	0.80	0.73	0.67
≥3 days	0.63	0.63	0.67	0.75	0.83	0.87	0.89	0.90	0.90	0.90	0.89	0.86	0.80	0.75
≥4 days	0.69	0.69	0.73	0.80	0.86	0.90	0.92	0.93	0.93	0.93	0.92	0.89	0.84	0.80
≥5 days	0.74	0.74	0.77	0.83	0.89	0.92	0.93	0.94	0.94	0.94	0.93	0.91	0.87	0.84
≥6 days	0.77	0.77	0.80	0.86	0.90	0.93	0.94	0.95	0.95	0.95	0.94	0.92	0.89	0.86
≥7 days	0.80	0.80	0.82	0.87	0.92	0.94	0.95	0.96	0.96	0.96	0.95	0.93	0.90	0.88
≥8 days	0.82	0.82	0.84	0.89	0.93	0.95	0.96	0.96	0.96	0.96	0.96	0.94	0.91	0.89
≥9 days	0.84	0.84	0.86	0.90	0.93	0.95	0.96	0.97	0.97	0.97	0.96	0.95	0.92	0.90
≥10 days	0.85	0.85	0.87	0.91	0.94	0.96	0.97	0.97	0.97	0.97	0.96	0.95	0.93	0.91

Reliability was low when children with at least one day lasting between one to three hours were included in analyses (36%–40%). PA measurement was more reliable when children with at least two days or greater were included in analyses. Measurement reliability values of at least 90% were achieved when the following thresholds were used to define which children were included in analyses: at least three days lasting at least eight hours per day, at least four or five days lasting at least six hours per day, and at least six days lasting at least five hours per day (90%, 90%, 92%, and 90% respectively). As defined by the Spearman- Brown prophecy formula, the most reliable measure of PA (97%) was achieved when children with at least nine or 10 days lasting from at least eight to at least 10 hours per day were included in analyses. A high reliability and sample size was achieved when children with at least two days lasting at least 10 hours per day were included in analyses (*n* = 6,528; reliability 86%).

### Gender

**Figure 1:**
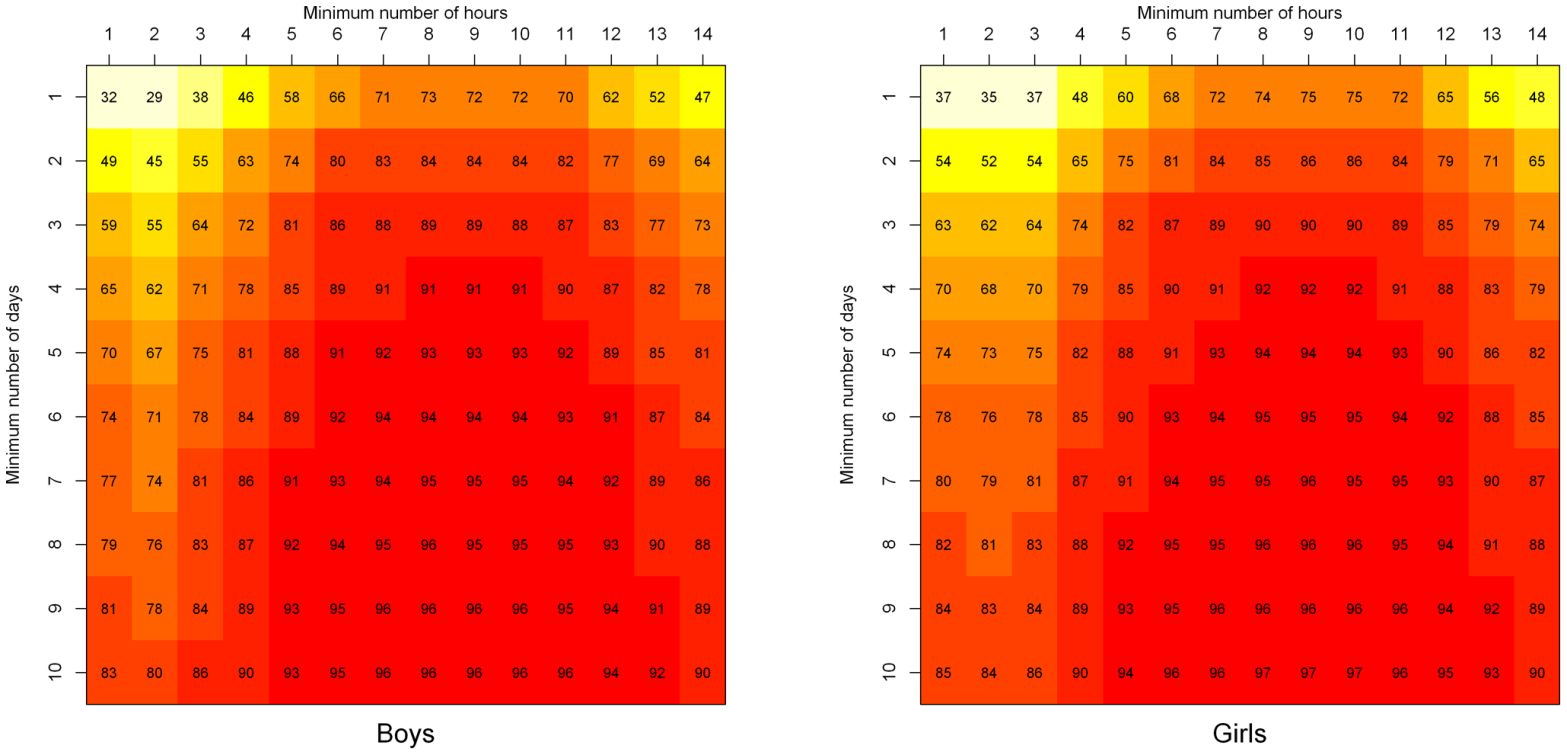
Reliability (%) Heatmaps for boys and girls by minimum daily wear time and wear days.

When reliability coefficients were calculated separately for boys and girls the results followed a similar trend in both genders to that found for the total sample (Figure 1). Reliabilities were again influenced by the minimum daily wear time and the minimum number of wear days. The reliability of PA measurement exhibited a minimal gender-related trend: measurement was slightly more reliable in girls than boys for nearly all combinations of minimum daily wear time and minimum number of wear days. The most reliable measure was achieved in boys when children with at least nine or 10 days lasting from at least seven to at least 11 hours per day (96%) were included in analyses and in girls with at least 10 days lasting from at least eight to at least 10 hours per day (97%).

### Inclusion of Weekend Days

A total of 2,414 singleton children (31.3% of all singletons returning data) returned an accelerometer that contained at least one day of data (≥ one hour) but no weekend day data. At least one weekend days’ worth of data (≥10 hours) was obtained from 5,290 singleton children (68.7% of all singletons returning data).

**Figure 2:**
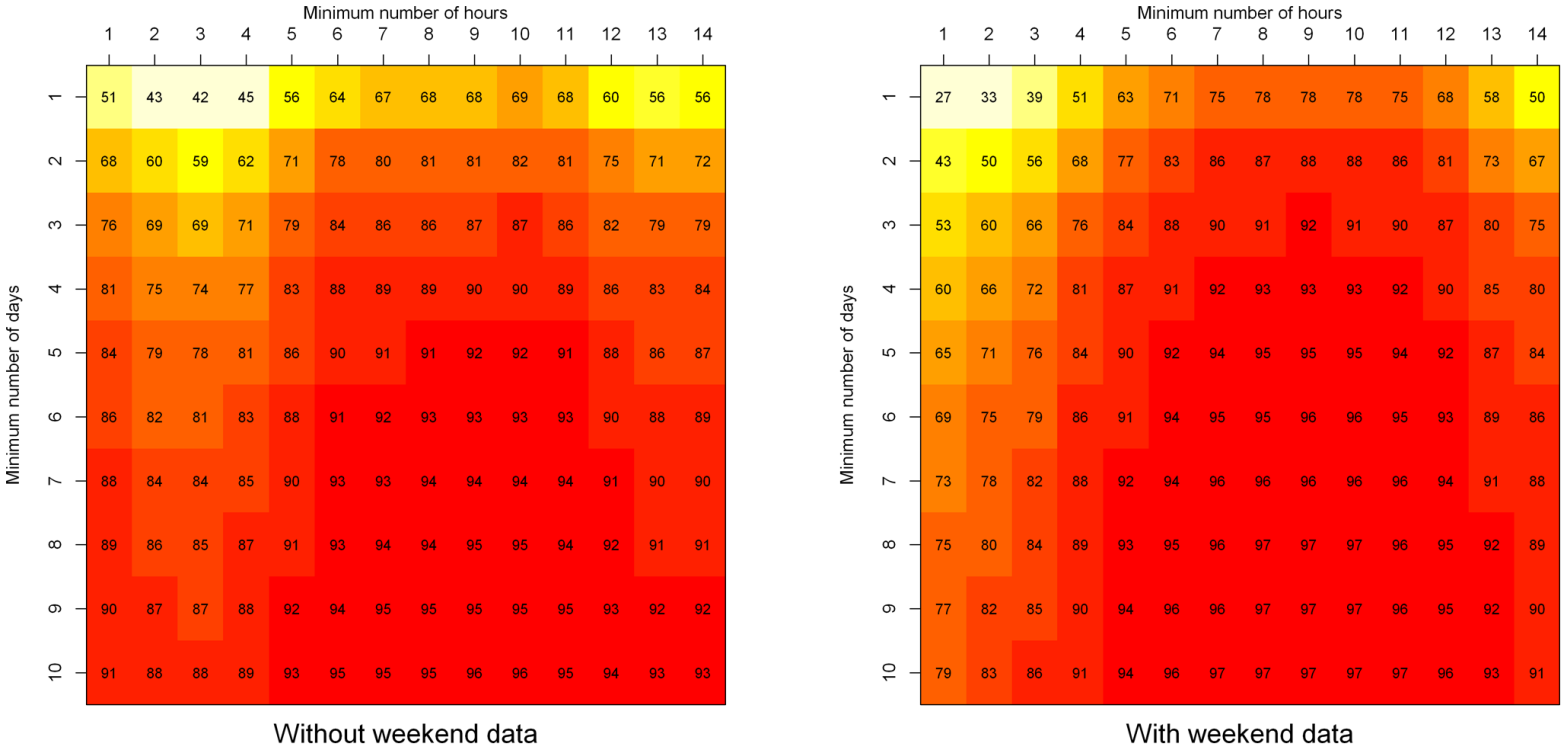
Reliability Heatmaps (%) for children with and without weekend data by minimum daily wear time and wear days.

Reliability coefficients increased as the minimum daily wear time and the minimum number of wear days increased in both children with and without weekend data. Reliabilities were slightly higher when only children with weekend data were included in analyses compared to children with only weekday data when wear time was defined as at least four hours per day up to 13 hours per day for all numbers of wear days (Figure 2). For example, when children with at least two days lasting at least 10 hours per day were included in analyses reliability was high in both children with and without weekend data but reached 82% in children with only weekday data compared to 88% in children with weekend data.

Reliabilities calculated when including only children with weekend data available were similar to those calculated when not purposely sampling children based on whether or not they had weekend data for all combinations of minimum daily wear times and number of wear days. For example, the reliability of mean daily cpm calculated from children with at least two days of data lasting at least 10 hours per day was 88% in children with at least one weekend day of data compared to 86% when not purposely sampling children based on weekend wear.

## Discussion

### Summary of Findings

A threshold of at least two days lasting at least 10 hours per day can be used to screen subjects who provide reliable estimates of PA in population studies of older primary school aged children. This threshold provided a high reliability and sample size (*n* = 6,528; reliability 86%). The use of this threshold also increases our ability to compare our findings with other studies, as the most common threshold previously used to define minimum daily wear time was at least 10 hours per day [3]–[5], [8]. The 80% reliability threshold has also been used by previous studies exploring the influence of varying the wear time threshold on the reliability of PA measurement [13], [16]. Both the minimum daily wear time and the minimum number of wear days required by each child for inclusion in analyses influenced the reliability of PA measurement. Reliabilities were similar for both genders, although measurement in girls was slightly more reliable than boys for nearly all combinations of minimum daily wear time and minimum number of wear days.

Reliabilities were slightly higher for children with weekend data compared to children with only weekday data. However, the purposeful sampling of children with at least one weekend day of data resulted in similar reliabilities for all wear time thresholds to those calculated when using the total sample independent of weekend wear. Therefore, our results suggest that population data should encourage the measurement of PA on weekend days, but the purposeful sampling of subjects which forces the inclusion of weekend data in *all* children is not necessary.

### Comparisons with Existing Research

Only two previous studies have explored the influence of varying the minimum daily wear time and the minimum number of days of data required from each child to be included in analyses. In contrast to our study, Mattocks *et al* [15] and Penpraze *et al* [13] found that the minimum daily wear time had less influence on reliability than the number of wear days. Penpraze *et al* [13] calculated the reliability of PA in 76 five to six year old Scottish children: measurement reliability remained relatively stable using at least three hours per day up to, but no more than, at least 10 hours per day. The authors reported lower reliabilities than those calculated in the present study [13]: defining wear time as at least seven days lasting at least 10 hours per day produced the highest reliability (80%, 95% CI = 70%, 86%). Penpraze *et al* [13] used a small geographically clustered sample and only included children with seven complete days of PA monitoring which has the potential to introduce bias in results.

Mattocks *et al* [15] found that the reliabilities remained constant using varying daily wear lengths (between seven to 10 hours) but whilst the number of wear days required per child to be included in analyses remained constant. The Avon Longitudinal Study of Parents and Children (ALSPAC) also reported lower reliabilities than our study using the same thresholds to define wear time: children with at least 12 days lasting at least seven hours per day of data produced the highest reliability (90%). However, the ALSPAC used a threshold of at least three days lasting at least 10 hours per day to define minimum wear time despite the reliability of measurement reaching only 70% when using data from children meeting this threshold.

A number of articles have examined the reliability of PA measurement using varying numbers of wear days without considering the influence of varying the daily wear length. The findings of these studies vary greatly, and are dependent on the age of the children and the study design. Studies have found that reliabilities of 80% are achieved when including children with a greater number of days of accelerometer data than calculated by our study. For example, at least five [10] to seven [13] wear days were required from preschool children (aged two to five years), four [16], [38] to seven [17], [38] wear days were required from children aged six to twelve years, and five [18] to nine [16] wear days were required from adolescents (aged 13 to 18 years). Despite these findings, other large-scale accelerometer studies in children have analysed data in children providing one day of accelerometer data [8], [18]. For example, the Trial Activity for Adolescent Girls study included children in analyses who provided at least one day of data lasting at least six hours [18].

To our knowledge, there have been no published studies exploring the influence of gender on the reliability of accelerometer-determined PA. Only a few studies have explored the influence of the distribution of wear days on the reliability of PA measurement in children [13]. In agreement with this study, Penpraze *et al* [13] found that the purposeful inclusion of children in analyses with weekend days had little effect on reliability estimates: using data provided by children with four days wear including a weekend day compared to using data provided by children with four weekday wear days only reduced reliability estimates from 84% to 82%. Mattocks *et al* [15] did not explore the influence of purposely sampling children based on weekend wear, but in agreement with other studies [8], [12], [20], they found that children’s PA differed on weekend days compared to weekdays.

Only one previous large-scale study has evaluated the influence of varying the number and distribution of accelerometer wear days on the reliability of population estimates of PA. In contrast to our study, McClain *et al* [23] found that stable estimates of population PA can be obtained from only one randomly selected day out of a possible sampled week in 2532 US adults (aged 20 years). However, in agreement with our study, they also found that the purposeful sampling of subjects which forces the inclusion of a weekend day is not necessary.

### Strengths and Limitations

This is the first study to explore the influence of varying the minimum daily wear time *and* the minimum number of days of accelerometer data on the reliability of PA measurement in a large-scale UK-wide study of children. It has been suggested that previous thresholds of minimum wear time may have been overestimated because of violations in the assumptions associated with the ICC formula [36], [39]. However, we have shown that high reliability values can be attained from children with a relatively small number of days and hours of wearing time. We have proposed a threshold that maximises both reliability and sample size whilst also taking into our account our ability to compare our findings with other studies. We are also confident that the study design, accelerometer protocol, and analytical methodologies employed here enable us to define a robust definition of wear time. Accelerometer data often follow a skew distribution, and it is important to account for this asymmetry to achieve the normality assumption required to correctly compute the ICC. Our ICC values also take into account the MCS survey and non-response weights. Uniquely, we have also explored the influence of gender and the distribution of wear days on the reliability of PA measurement. In doing so, we used data from a large, contemporary, socially- and ethnically- diverse cohort of children from all four UK countries.

Our proposed wear time threshold may not be applicable for use in different ages [16]. PA levels vary according to age, and children’s PA is very different to adult’s PA in many respects [3]; it is therefore unlikely that without further research the findings of this study can be used in adolescent or adult populations. Reliability values may also be dependent on the derived PA outcome variable. It has been widely documented that the use of different thresholds to define activity intensities limits the ability for researchers to make reliable comparisons of moderate to vigorous PA levels between studies, and at present there is still no consensus on the best threshold to use [40]. However, studies have found similar reliability values for PA measurement when accelerometer data were expressed as cpm or as the percentage of time in different activity intensities [13], [41].

### Recommendations for Study Practice and further Research

It is important that researchers using accelerometer data only analyse data from children that meet a pre-defined wear time threshold. Using the proposed threshold will enhance quality control processes by ensuring that only children that provide enough data to reliably estimate weekly PA are used in analyses without compromising sample size. If population studies do not screen accelerometer data prior to processing this may lead to unreliable estimates of children’s habitual activity levels. The proposed threshold is appropriate for use in boys and girls, although studies using samples of only girls may be able to use a less stringent definition than studies including both genders. It is important that subjects are asked to wear their accelerometer over an entire day, and that both weekdays and weekend days are requested in the monitoring period. However, the purposeful inclusion of children with weekend data in analyses is not necessary.

Future research should be aimed at calculating whether the proposed threshold is applicable across different age groups and in studies deriving different PA outcome variables. Furthermore, this study suggests that the inclusion of data from children with at least two days of accelerometer data (at least 10 hours/day) out of a possible seven day monitoring period provides a reliable estimate of population-based estimates of PA. Further research is required to determine whether this is applicable in studies that ask children to wear their monitor for only two days. Bias may be introduced when data from children with only two wear days are included in analyses, especially if these days are not randomly sampled from a possible seven day week. Although beyond the scope of this study, the removal of children that do not meet the wear time threshold may be dealt with through imputation methods [42], and future research is needed to explore such approaches to adjust for potential bias introduced by removing unreliable data.

### Conclusions

It is important for population-based studies to integrate a core set of quality control procedures prior to deriving activity outcome variables: this should include the screening of data using a wear time threshold. Using a threshold of at least two days lasting at least 10 hours per day will enhance data quality. This threshold is applicable in 7–8 year olds and in population-based studies that monitor children over a full week including the weekend. It is unnecessary to only include children with weekend data in analyses.

## References

[1] Baquet G, Stratton G, Van PE, Berthoin S (2007) Improving physical activity assessment in prepubertal children with high-frequency accelerometry monitoring: a methodological issue. PrevMed 44: 143–147. 10.1016/j.ypmed.2006.10.004 17157370

[2] Rowlands AV (2009) Acceleromter Assessment of Physical Activity in children: An update. Pediatric Exercise Science 19: 252–266. 10.1123/pes.19.3.252 18019585

[3] Troiano RP, Berrigan D, Dodd KW, Masse LC, Tilert T, et al (2008) Physical activity in the United States measured by accelerometer. MedSciSports Exerc 40: 181–188. 10.1249/mss.0b013e31815a51b3 18091006

[4] Riddoch CJ, Bo AL, Wedderkopp N, Harro M, Klasson-Heggebo L, et al (2004) Physical activity levels and patterns of 9- and 15-yr-old European children. MedSciSports Exerc 36: 86–92. 10.1249/01.MSS.0000106174.43932.92 14707773

[5] Riddoch CJ, Mattocks C, Deere K, Saunders J, Kirkby J, et al (2007) Objective measurement of levels and patterns of physical activity. ArchDisChild 92: 963–969. 10.1136/adc.2006.112136 PMC2083612 17855437

[6] Meeker WQ, Escobar LA (1998) Statistical Methods for Reliability Data. New Jersey: Wiley.

[7] Thompson AM, Campagna PD, Durant M, Murphy RJ, Rehman LA, et al (2009) Are overweight students in Grades 3, 7, and 11 less physically active than their healthy weight counterparts? IntJPediatrObes 4: 28–35. 10.1080/17477160802170050 19205979

[8] Owen CG, Nightingale CM, Rudnicka AR, Cook DG, Ekelund U, et al (2009) Ethnic and gender differences in physical activity levels among 9–10-year-old children of white European, South Asian and African-Caribbean origin: the Child Heart Health Study in England (CHASE Study). IntJEpidemiol 38: 1082–1093. 10.1093/ije/dyp176 PMC2720395 19377098

[9] Esliger DW, Tremblay MS, Copeland JL, Barnes JD, Huntington GE, et al (2010) Physical activity profile of Old Order Amish, Mennonite, and contemporary children. MedSciSports Exerc 42: 296–303. 10.1249/MSS.0b013e3181b3afd2 19927029

[10] Taylor RW, Murdoch L, Carter P, Gerrard DF, Williams SM, et al (2009) Longitudinal study of physical activity and inactivity in preschoolers: the FLAME study. MedSciSports Exerc 41: 96–102. 10.1249/MSS.0b013e3181849d81 19092702

[11] van Sluijs EM, Skidmore PM, Mwanza K, Jones AP, Callaghan AM, et al (2008) Physical activity and dietary behaviour in a population-based sample of British 10-year old children: the SPEEDY study (Sport, Physical activity and Eating behaviour: environmental Determinants in Young people). BMCPublic Health 8: 388. 10.1186/1471-2458-8-388 PMC2605463 19014571

[12] Nilsson A, Anderssen SA, Andersen LB, Froberg K, Riddoch C, et al (2009) Between- and within-day variability in physical activity and inactivity in 9- and 15-year-old European children. ScandJMedSciSports 19: 10–18. 10.1111/j.1600-0838.2007.00762.x 18248534

[13] Penpraze V, Reilly J, MacLean C, Montgomery C, Kelly L, et al (2006) Monitoring of physical activity in young children: how much is enough? Pediatric Exercise Science 18: 483–491. 10.1123/pes.18.4.483 39152609

[14] Hinkley T Fau - O'Connell E, O'Connell E Fau - Okely AD, Okely Ad Fau - Crawford D, Crawford D Fau - Hesketh K, Hesketh K Fau - Salmon J, et al. Assessing volume of accelerometry data for reliability in preschool children. 10.1249/MSS.0b013e3182661478 22776873

[15] Mattocks C, Ness A, Leary S, Tilling K, Blair SN, et al (2008) Use of accelerometers in a large field-based study of children: protocols, design issues, and effects on precision. JPhysActHealth 5 Suppl 1S98–111. 10.1123/jpah.5.s1.s98 18364528

[16] Trost SG, Pate RR, Freedson PS, Sallis JF, Taylor WC (2000) Using objective physical activity measures with youth: how many days of monitoring are needed? MedSciSports Exerc 32: 426–431. 10.1097/00005768-200002000-00025 10694127

[17] Treuth MS, Sherwood NE, Butte NF, McClanahan B, Obarzanek E, et al (2003) Validity and reliability of activity measures in African-American girls for GEMS. MedSciSports Exerc 35: 532–539. 10.1249/01.MSS.0000053702.03884.3F 12618587

[18] Murray DM, Stevens J, Hannan PJ, Catellier DJ, Schmitz KH, et al (2006) School-level intraclass correlation for physical activity in sixth grade girls. MedSciSports Exerc 38: 926–936. 10.1249/01.mss.0000218188.57274.91 PMC2034369 16672847

[19] Gidlow CJ, Cochrane T, Davey R, Smith H (2008) In-school and out-of-school physical activity in primary and secondary school children. JSports Sci 26: 1411–1419. 18942001 10.1080/02640410802277445

[20] Rowlands AV, Pilgrim EL, Eston RG (2008) Patterns of habitual activity across weekdays and weekend days in 9–11-year-old children. PrevMed 46: 317–324. 10.1016/j.ypmed.2007.11.004 18162187

[21] Masse LC, Fuemmeler BF, Anderson CB, Matthews CE, Trost SG, et al (2005) Accelerometer data reduction: a comparison of four reduction algorithms on select outcome variables. MedSciSports Exerc 37: S544–S554. 10.1249/01.mss.0000185674.09066.8a 16294117

[22] Tucker J, Welk G (2010) Accelerometer Data Processing in NHANES 2005–2006: Evaluation of Physical Activity Compliance Criteria. American College of Sports Medicine 2010 Annual Meeting.

[23] McClain JJ, Dodd KW, Berrigan D, Troiano RP (2010) How many accelerometer days are needed for stable population and individual weekley activity estimates? American College of SPorts Medicine Annnual Meeting.

[24] Esliger DW, Copeland JL, Barnes JD, Tremblay MS (2005) Standardizing and Optimizing the Use of Accelerometer Data for Free-Living Physical Activity Monitoring. JPhysActHealth 3: 366–383.

[25] Plewis I (2007) Millennium Cohort Study: technical report on sampling 4th edition. London: Institute of Education.

[26] Janz KF (1994) Validation of the CSA accelerometer for assessing children's physical activity. MedSciSports Exerc 26: 369–375. 8183103

[27] Trost SG, Way R, Okely AD (2006) Predictive validity of three ActiGraph energy expenditure equations for children. MedSciSports Exerc 38: 380–387. 10.1249/01.mss.0000183848.25845.e0 16531910

[28] Plasqui G, Westerterp KR (2007) Physical activity assessment with accelerometers: an evaluation against doubly labeled water. Obesity(SilverSpring) 15: 2371–2379. 10.1038/oby.2007.281 17925461

[29] Team RCD (2010) R: A language and environment for statistical computing website. Vienna, Austria: R Foundation for Statistical Computing.

[30] Spearman CE (1904) 'General Intelligence' objectively determined and measured. American Journal of Psychology 5: 201–293.

[31] Spearman CE (1904) Proof and measurement of association between two things. American Journal of Psychology 5: 72–101. 3322052

[32] Box GEP, Cox DR (1964) An Analysis of Transformations. Journal of the Royal Statistical Society: Series A (Methodological): 211–252.

[33] Venables WN, Ripley BD (2002) Modern Applied Statistics with S. New York: Springer Science+Business Media, Inc.

[34] Pinheiro JC, Bates DM (2000) Mixed-Effects Models in S and S-Plus. New York: Springer Verlag.

[35] Fletcher TD (2012) R package ICC1.lme: Intraclass Correlation Coefficient from a Mixed-Effects Model. R package version 2.2 ed.

[36] Baranowski T, Masse LC, Ragan B, Welk G (2008) How many days was that? We're still not sure, but we're asking the question better! MedSciSports Exerc 40: S544–S549. 18562972 10.1249/MSS.0b013e31817c6651 PMC2739114

[37] Carmines EG, Zeller RA (1979) Reliability and validity assessment; Sage, editor. California: Sage Publications.

[38] Nader PR, Bradley RH, Houts RM, McRitchie SL, O'Brien M (2008) Moderate-to-vigorous physical activity from ages 9 to 15 years. JAMA 300: 295–305. 18632544 10.1001/jama.300.3.295

[39] Matthews CE, Hagstromer M, Pober DM, Bowles HR (2012) Best practices for using physical activity monitors in population-based research. Med Sci Sports Exerc 44: S68–76. 22157777 10.1249/MSS.0b013e3182399e5b PMC3543867

[40] Corder K, Brage S, Ekelund U (2007) Accelerometers and pedometers: methodology and clinical application. CurrOpinClinNutrMetab Care 10: 597–603. 10.1097/MCO.0b013e328285d883 17693743

[41] Janz KF, Witt J, Mahoney LT (1995) The stability of children's physical activity as measured by accelerometry and self-report. MedSciSports Exerc 27: 1326–1332. 8531633

[42] Catellier DJ, Hannan PJ, Murray DM, Addy CL, Conway TL, et al (2005) Imputation of missing data when measuring physical activity by accelerometry. MedSciSports Exerc 37: S555–S562. 10.1249/01.mss.0000185651.59486.4e PMC2435061 16294118

